# Enlarged Spleen Treated by Ergot Hypodermatically

**Published:** 1886-03

**Authors:** C. L. Gwyn

**Affiliations:** Galveston, Texas


### REPORT OF CASES OF ENLARGED SPLEEN TREATED BY HYPODERMIC INJECTIONS OF ERGOTINE. (FROM MEMORANDA.)

*By C. L. Gwyn, M. D., Galveston, Texas.*

For Daniel's Texas Medical Journal.

Case 1. Tommy Clarke, Texan, age 13 years, from a coast country. Tertian intermittent fever, duration of disease about five years, with intermissions of three to five months; spleen chronically engorged and enlarged, enlarged beyond and below the umbilicus. Used xv gtt solution of ergotine, five times over the spleen, at intervals of four or five days. Discharged cured July, 1878.

Case 2. — Long, Harris county, age 12 years, intermittent fever; general anæmia and enlarged spleen. His family were unable to give any definite account of duration of disease. Quinine, arsenic, iron, and tonics generally, afforded but temporary relief; used solution ergotine, xv gtt hypodermically, once every week for four weeks. Discharged cured September, 1878. This patient is still living with his parents, tenants in the neighborhood, and has not been unwell a day since discharged.

Case 3. Louis Weaver, Texan, aged 12 years, malarial purpura hemorrhagica, with epistaxis, the hemorrhage only appearing during febrile exacerbation. Gave quinine and injected ergotine; gave three injections of xx gtt three weeks apart for his enlarged spleen. Discharged cured September, 1879. This patient during the past year had an attack of intermittent fever, which yielded readily to quinine.

These cases were of sallow complexion, so much so that their friends accused them of being dirt-eaters. The sallowness has all disappeared, and they are now as hearty and ruddy as well can be.

I used Bonjean's ergotine, rubbed down with just sufficient glycerine to make it fluid enough to use with the hypodermic syringe, and supposed that xx gtt would represent about three grains.

These cases were treated in 1878 79–80. Since then I have used injections of ergotine, and liq. ergotæ purificatus in some twenty odd cases, and have had but one abscess to follow its use, and it has caused intense pain in but one case, and in every case successfully.

The use of ergot in the above manner, I believe, was first suggested by Da Costa.

I do not wish it understood that I abandoned treatment by quinine and tonics while using the ergotine, but that these cases proved rebellious to treatment until the hypodermic needle was used.

